# Synergistic and antagonistic interactions of oxybenzone and ocean acidification: new insight into vulnerable cellular processes in non-calcifying anthozoans

**DOI:** 10.3389/fphys.2023.1332446

**Published:** 2024-01-11

**Authors:** Michael B. Morgan, Jacob Williams, Barrett Breeze, Nicholas English, Nathaniel Higdon, Kirt Onthank, Dominic F. Qualley

**Affiliations:** ^1^ Department of Biology, Berry College, Mount Berry, GA, United States; ^2^ Department of Chemistry and Biochemistry, Berry College, Mount Berry, GA, United States; ^3^ Department of Biology, Walla Walla University, College Place, WA, United States

**Keywords:** ocean acidification, oxybenzone, gene expression profiles, biomarkers, multiple stressors, *in silico* modeling, synergistic response, antagonistic response

## Abstract

Cnidarians face significant threats from ocean acidification (OA) and anthropogenic pollutants such as oxybenzone (BP-3). The convergence of threats from multiple stressors is an important area to investigate because of potential significant synergistic or antagonistic interactions. Real-time quantitative PCR was performed to characterize the expression profiles of twenty-two genes of interest (GOI) in sea anemones (*Exaiptasia diaphana)* exposed to one of four treatments: 1) 96 h of OA conditions followed by a 4 h exposure to 20 ppb BP-3; 2) Exposure to 4 h 20 ppb BP-3 without 96 h of OA; 3) Exposure to 96 h of OA alone; or 4) laboratory conditions with no exposure to BP-3 and/or OA. These 22 GOIs represent cellular processes associated with proton-dependent transport, sodium-dependent transport, metal cation binding/transport, extracellular matrix, amino acid metabolism/transport, immunity, and/or steroidogenesis. These 22 GOIs provide new insight into vulnerable cellular processes in non-calcifying anthozoans exposed to OA and BP-3. Expression profiles were categorized as synergistic, antagonistic, or additive of BP-3 in the presence of OA. Two GOIs were synergistic. Fifteen GOIs were antagonistic and the remaining five GOIs were additive in response to BP-3 in acidified seawater. A subset of these GOIs appear to be candidate biomarkers for future *in situ* investigations. In human health, proton-dependent monocarboxylate transporters (MCTs) are promising pharmacological targets and recognized as potential biomarkers. By comparison, these same MCTs appear to be targets of xenobiotic chemical pollutants in cnidarian physiology. In the presence of BP-3, a network of collagen synthesis genes are upregulated and antagonistic in their expression profiles. Cytochrome b561 is a critical protein required for collagen synthesis and *in silico* modeling demonstrates BP-3 binds in the pocket of cytochrome b561. Understanding the underlying molecular mechanisms of “drug-like” compounds such as BP-3 may lead to a more comprehensive interpretation of transcriptional expression profiles. The collective antagonistic responses of GOIs associated with collagen synthesis strongly suggests these GOIs should be considered candidate biomarkers of effect. GOIs with synergistic and additive responses represent candidate biomarkers of exposure. Results show the effects of OA and BP-3 are interactive with respect to their impact on cnidarians. This investigation offers mechanistic data that supports the expression profiles and underpins higher order physiological responses.

## Introduction

Ocean acidification is a global concern characterized by the increase in the acidity of the world’s oceans due to the uptake of anthropogenic carbon dioxide (CO_2_) from the atmosphere. Atmospheric CO_2_ concentrations have risen from pre-industrial levels of approximately 275 ppm (Macfarling Meure et al., 2006) to over 400 ppm (Dunn et al., 2020), with nearly a third of all annual production of anthropogenic production of CO_2_ being absorbed by the world oceans (Doney et al., 2009). The resulting drop in pH of marine environments poses significant threats to a broad range of marine organisms (Fabry et al., 2008). Meta-analysis of extant taxa responding to OA reveals similar trends in the negative responses of diverse phyla including cnidarians, mollusks, echinoderms, crustaceans, and bony fish (Kroeker et al., 2013; Wittmann and Pörtner, 2013; Medeiros and Souza, 2023). For calcifying organisms, OA leads to the reduction in the saturation state of carbonate ions, which hinders the calcification process. For corals, OA critically impacts growth and survival (Hoegh-Guldberg et al., 2007). A majority of previous cnidarian investigations have focused on calcification, skeletal density, and/or early life stages (Moya et al., 2012; Putnam et al., 2013; Ramos-Silva et al., 2013; Traylor-Knowles and Connelly, 2017). Some phyla such as Cnidaria and Mollusca also have taxa which are not calcifiers, so investigations not directly linked to calcification are also needed for a broader understanding of OA impact. Sea anemones in the genus *Exaiptasia* are ideal representatives of non-calcifying anthozoans that are easily cultured in the laboratory and utilized for ecotoxicological studies. Representatives of the genus are found worldwide in tropical marine ecosystems, have the same body plan as scleractinian corals, and are capable of establishing/maintaining symbiotic relationships with zooxanthellae (Sunagawa et al., 2009; Main et al., 2010; Schlesinger et al., 2010; Howe et al., 2012; Siddiqui et al., 2015; Duckworth et al., 2017).

Tropical coastal marine ecosystems are particularly notable for a number of diverse features. Coastal upwellings are locations that increase the probability of OA conditions (Manzello, 2010; Murray et al., 2015). Concurrently, anthropogenic activity along coastlines displays a concentration gradient from highest to lowest (nearshore to offshore, respectively) and is routinely driven by seasonal climatic activity (Dachs et al., 1999; Gray et al., 2002; Yamashita and Tanoue, 2003; Fabry et al., 2008; Gavio et al., 2010; Linsmayer et al., 2020; Zhu et al., 2022). As a consequence, coastal marine ecosystems are exposed to a diversity of anthropogenic stressors and these environments are increasingly becoming “sinks” for complex mixtures of anthropogenic chemicals originating from diverse sources (Peters et al., 1997; Atkinson et al., 2003; Wurl and Obbard, 2004; Fent et al., 2006; Schiedek et al., 2007; Halpern et al., 2008; Jones, 2010; Burns and Brinkman, 2011; Dafforn et al., 2011; Vidal-Dorsch et al., 2012; French et al., 2015; Pie et al., 2015; Wear and Thurber, 2015; Downs et al., 2016; Archer et al., 2017; Law, 2017; Worm et al., 2017; Lebreton et al., 2018; Mitchelmore et al., 2019). As a consequence, marine organisms in coastal marine ecosystems face significant threats from diverse anthropogenic chemical pollutants.

Oxybenzone (BP-3), the active ingredient in sunscreen, is just one of many xenobiotics present in the marine environment (Vidal-Dorsch et al., 2012). It is estimated that 14,000 tons of sunscreens enter the oceans annually from beachgoers and domestic sewage effluent, and there is a growing body of evidence of BP-3 acts as an endocrine disruptor on cnidarians (Downs et al., 2016; Mitchelmore et al., 2019; Mitchelmore et al., 2021; Morgan et al., 2022).

The convergence of threats from OA and chemical pollution is an important area of investigation because of potential significant interactions between anthropogenic pollutants and OA conditions (Nikinmaa, 2013). The natural environment is a mixture of multiple stressors causing mixed effects and challenging organisms to acclimatize and/or adapt to physiologically demanding conditions (Evans and Hofmann, 2012; Stillman et al., 2015; Deidda et al., 2021). For example, heavy metal toxicity is known to be exacerbated in sea anemones experiencing OA (Siddiqui et al., 2015; Duckworth et al., 2017). Currently, there are knowledge gaps in cnidarian transcriptional responses to BP-3 within an environment of OA. This study seeks to characterize patterns of transcription in genes of interest (GOIs) in sea anemones responding to BP-3 and acidic seawater.

## Materials and methods

### CO_2_-acidified conditions

Two 38 L glass aquaria were filled with raw seawater obtained from the Rosario Beach Marine Laboratory seawater system, which is in turn pumped from Rosario Bay, Fidalgo Island, Washington, US. Seawater was allowed to equilibrate to room temperature, approximately 21°C, before being added to the aquaria. Temperature and pH of each aquarium was monitored using an Open Acidification Tank Controller unit (Onthank et al., 2023). The pH of the treatment aquarium was adjusted lower by bubbling pure CO_2_ into a bubble stone from a solenoid-equipped CO_2_ regulator on a 2.3 kg high pressure CO_2_ cylinder and controlled by the Open Acidification Tank Controller. A pH of 7.5 was the target pH for the treatment aquarium for two reasons: 1) the pH of 7.5 is approximately 0.3 units lower than present day pH of seawater found in Rosario Bay (Onthank et al., 2021), and 2) the pH of 7.5 is below BP-3′s pKa of 7.6 (Fontanals et al., 2010). The pH of the control aquarium was not controlled. The temperature of the aquaria was not controlled and allowed to match the room temperature. Independent measurements of pH were taken of each aquarium daily using the m-cresol purple spectrophotometric method (Dickson et al., 2007) as modified by Culler-Juarez and Onthank (2021). Total pH values were calculated from absorbance values in R using the “specpH” function found the in OTools package version 22.08.01 (https://github.com/KirtOnthank/Otools). Total alkalinity of each aquarium was measured on day 1 and day 4 using the open-cell titration method (Dickson et al., 2007) and alkalinity was calculated from titration data in R using the “at” function in the seacarb package version 3.2.14 (Gattuso et al., 2015). Salinity was measured daily using a Vernier salinity probe (SAL-BTA). Temperature was measured daily using a VWR precision liquid-in-glass thermometer. Partial pressure of carbon dioxide in each aquarium was calculated each day from daily pH, salinity, and temperature measurements, and from average of total alkalinity measurements for each aquarium in R using the “carb” function from in the “seacarb” function version 3.2.14 (Gattuso et al., 2015). All raw data and code to calculate derived values are available in a repository on Zenodo (https://doi.org/10.5281/zenodo.8011603).

### Toxicant exposure

Sea anemones (*Exaiptasia diaphana)* were purchased from a supplier (Carolina Biological Supply, Burlington, NC, United States) and acclimated to laboratory conditions (recirculating natural seawater at 21°C, ambient laboratory lighting, and 29 ppt salinity) for 1 month prior to beginning the experiment. All BP-3 treatments were nominal concentrations for 4 h in 1L seawater under ambient laboratory lighting during the mid-summer season. The 20 ppb concentration was chosen to induce responses and does not necessarily reflect environmental conditions. Previous studies have demonstrated a toxicant concentration of 20 ppb will induce detectable differential gene expression after 4 h of exposure (Morgan et al., 2001; Morgan and Snell, 2002; Morgan and Snell, 2006; Morgan et al., 2012; Morgan et al., 2022). Variations in environmental conditions can influence the toxicity of xenobiotics (Xin et al., 2021). A 4 h exposure was arbitrarily chosen as an initial timeframe since environmental BP-3 is associated with anthropogenic beach activity. The 96 h timeframe of OA exposure was chosen since some coastal upwellings are known to have periodicities of fluctuation (Murray et al., 2015; Rodriguez-Ruano et al., 2023). Dynamic environments such as an acute 96 h exposure of OA followed by a 4 h pulse of BP-3 will challenge organisms to adapt to physiologically demanding conditions (Evans and Hofmann, 2012). BP-3 was solubilized in acetone at 100 μL/L prior to dilution in seawater. Anemones were divided into four treatments: 1) 96 h of OA conditions followed by a 4 h exposure to 20 ppb oxybenzone (BP-3); 2) Exposure to 4 h 20 ppb BP-3 without 96 h of OA; 3) Exposure to 96 h of OA alone; or 4) laboratory conditions without exposure to BP-3 and/or OA conditions. The 96 h timeframe was chosen because there is evidence of different patterns of responses between short- and long-term CO_2_ exposures (Inbakandan, 2016; Medeiros and Souza, 2023).

### Selecting candidate genes of interest

A plethora of studies have investigated OA effects on calcifying marine organisms. Meta-analysis of extant taxa responding to OA reveals similar trends in the negative responses of diverse phyla including cnidarias, mollusks, echinoderms, crustaceans, and bony fish (Kroeker et al., 2013; Wittmann and Pörtner, 2013). Some phyla (i.e., Cnidaria and Mollusca) also have taxa which are not calcifiers (i.e., anemones and octopus, respectively). Hence, there is value in investigating responses to OA in non-calcifying taxa within calcifying phyla. In this investigation, genes on a cnidarian stress EST library were compared to an *O. rubescens* transcriptome from octopus exposed to OA conditions (data unpublished). Previous studies on model eukaryotes have shown that only 5%–10% of their genes respond to stressed conditions (Adams et al., 2000; Gasch et al., 2000; Hill et al., 2000; Goldstone, 2008). Since both phyla contain calcifying and non-calcifying taxa, this comparison offers the opportunity to identify genes that are representative of stress responses of non-calcifying organisms responding to xenobiotic chemicals and OA conditions. Candidate genes of interest (GOIs) were identified from homologous proteins (BLASTX analysis: nr database, standard parameters) found in both the cnidarian stress EST library and the *O. rubescens* transcriptome data. Subsets of ESTs from the cnidarian stress library have previously been used to characterize other cnidarian stress responses (Morgan et al., 2012; Morgan et al. 2015; Morgan et al. 2022)

### Reverse transcription reactions

Six to 10 anemones were pooled and homogenized for each treatment. Total RNA was isolated using Trizol (Invitrogen, United States). RNA concentration and quality were assessed using a NanoDrop ND-1000 spectrophotometer (Thermo Fisher Scientific, United States). Total RNA from pooled samples for each treatment was DNase I digested (New England BioLabs, United States), purified by chloroform/phenol extraction, and then reverse transcribed. First stand synthesis used SuperScript IV (Invitrogen, United States) along with random hexamers and oligo-dT primers to reverse transcribe 1 μg of total RNA. Reverse transcription conditions were 1 h at 37°C, followed by 1 min at each temperature between 42°C and 50°C.

### Quantitative real-time PCR

A QuantStudio 7 Flex Real-Time PCR system (Applied Biosystems, United States) used a SYBR Green-based assay to perform qPCR. A 1/100 dilution of first-strand synthesis reactions were used as templates for all qPCR reactions. Primers for each GOI were created using Primer3 (https://primer3.org). Components for each 20 µL qPCR reaction included: 10 µL Luna^®^ universal qPCR Mix (New England BioLabs, United States), 2.5 µL Forward primer (10 µM stock), 2.5 µL Reverse primer (10 µM stock), 2.5 µL dH_2_O, 2.5 µL sample. Thermocycling conditions were 1 cycle at 95°C for 1 min; 40 cycles of 95°C for 15 s and then 60°C for 30 s; and concluding with 1 cycle of melt curve analysis. Three to four technical replicate reactions were used for analyzing the relative expression of each GOI. Because of a consistent expression pattern, *RPL11* was used as the qPCR reference gene (Kenkel et al., 2011). Melt-curve analysis, primer efficiencies, and gel electrophoresis confirmed specificity of priming. Replicate Cq values were averaged to determine ∆Cq and ∆∆Cq for each treatment and GOI. All ∆Cq and ∆∆Cq values are based on the consistent expression of the endogenous reference control gene (*RPL11)* across all treatments. The ∆∆Cq method was used to determine the differences between targeted GOIs and a single reference gene (Bustin et al., 2009). Two-way ANOVA was performed on Log 2 transformed relative quantity data to determine the significant of the main effects and interactions. All *p*-values were adjusted using the Benjamini–Hochberg FDR correction.

### *in silico* molecular modelling

Human protein homologs have previously been used as substitutes for cnidarian proteins in molecular modelling (Khalturin et al., 2018). Some cnidarian proteins have such significant homology to human proteins that they are even capable of stimulating human responses (Dunn et al., 2006; Wood-Charlson and Weis, 2009; Duffy and Frank, 2011; Dani et al., 2014; Brennan et al., 2017; Mansfield et al., 2017). The most similar vertebrate structures were used for selecting the Protein Databank (PDB) files to be used for initial docking experiments. In the case of MCT1, cryo-EM structures of the homologous human protein in both inward-facing and outward-facing conformations are available in the PDB (PDB IDs 7cko and 7ckr, respectively). These structures were used for docking after removal of water and ligand molecules. The same treatment was applied to cytochrome b561, using 4o79 for the construct facing inward (cytosolic side) and 4o7g for the construct facing outward (non-cytosolic side). For MCTs 2 and 3, models were constructed using *H. sapiens* sequences corresponding to MCT2 (GenBank: AF049608.1) and MCT3 (GenBank: U81800.1) due to the lack of information on the cnidarian sequences. The cnidarian sequence was used to model MCT10 (GenBank: KXJ27932.1). All models of MCT2, 3, and 10 were constructed with the SWISS-MODEL web server (https://swissmodel.expasy.org) (Bertoni et al., 2017; Bienert et al., 2017; Waterhouse et al., 2018; Studer et al., 2020; Studer et al., 2021) using 7cko for inward-facing constructs and 7ckr for outward-facing constructs as templates. Ligands were created in Avogadro (Hanwell et al., 2012) and energy-minimized prior to docking. Docking was performed using AutoDock Vina (Trott and Olson, 2010); the search space was restricted to the known ligand binding site. Docking included protonated and deprotonated ligands. After docking, the results were analyzed in UCSF Chimera (Pettersen et al., 2004).

### Statistical analysis

After assumptions were tested by Levene’s test for homogeneity of variance across groups and Shapiro-Wilk Normality test, a two-way ANOVA was conducted for each gene to determine the significance of the main effects of OA and BP-3, as well as their potential interactions. The *p*-values obtained from these ANOVAs underwent an adjustment using the Benjamini–Hochberg False Discovery Rate (FDR) correction method to correct for multiple comparisons.

Genes were classified as “additive” where the interaction *p*-value exceeded 0.05, suggesting a non-significant interaction effect on gene expression between OA and BP-3. In this scenario, the combined effect of OA and BP-3 on the slope of gene expression can be interpreted as the summation of their independent effects. Genes were categorized as “synergistic” when both the slopes of interaction plots were either both positive or both negative with control OA being less steep than experimental OA exposure. In the context of this study, synergistic effects denote a scenario where the combined influence of OA and BP-3 on gene expression is greater than what would be anticipated from the sum of their individual effects. Lastly, “antagonistic” effects were identified for genes showing opposing slopes in the interaction plots, implying a counteracting influence between OA and BP-3 exposures on the anemone’s gene expression. Data and code underlying these analyses is available at https://zenodo.org/doi/10.5281/zenodo.10056756.

## Results

Acclimated anemones were separated into two groups (control and CO_2_-acidified treatment). At the beginning of the 96 h of exposure to acidified seawater the starting pH was 8.25. At the end of 96 h of exposure to acidified seawater, pCO_2_, pH, alkalinity, salinity, and temperature was recorded (see Table 1).

**TABLE 1:** Experimental conditions at the end of exposure to 96hrs of acidified seawater.

Treatment	pCO_2_ ( μ atm)	pH	Alkalinity ( μ mol kg^-1^)	Salinity (PSU)	Temperature (C)
Control	247 ± 47	8.348 ± 0.066	2048 ± 6	29.5 ± 0.1	21.3 ± 0.2
Elevated CO_2_	2045 ± 45	7.499 ± 0.009	2040 ± 1	29.5 ± 0.1	21.4 ± 0.1

### Genes of interest

This investigation quantified expression of 22 GOIs: 2-oxoglutarate and iron-dependent oxygenase domain-containing protein 2 (2OG-Fe (II)), 17β-hydroxysteroid dehydrogenase type 14 (17βHSD14), 17β-hydroxysteroid dehydrogenase type 12 (17βHSD12), Collagen alpha-2 (I) chain (COL1A2), Collagen alpha-1 (IV) chain (COL4A1), complement component C3 (C3), Cytochrome b561(CYB561), Glutamine synthetase (GLUL), Hephaestin (Heph), Integrin beta-PS (MYS), low affinity copper uptake protein 2 (SLC31A2-1), Monocarboxylate Transporter 2 (MCT2, SLC16A7), Monocarboxylate Transporter 3 (MCT3, SLC16A3), Monocarboxylate Transporter 10 (MCT10, SLC16A10), ornithine aminotransferase, mitochondrial-like (OAT), Serotransferrin-like (TF), Sodium/calcium exchanger 3 (SLC8A3), Soma ferritin-like (FTH1), Tauropine dehydrogenase (TaDH), Tenascin-R (TNR) has a FReD (containing a fibrinogen domain) and is also known as ryncolin-4), Transient receptor potential cation channel subfamily A member 1-like (TRPA1), Zinc transporter ZIP14 (SLC39A14). These GOIs are associated with proton-dependent transport, sodium-dependent transport, metal cation binding/transport, extracellular matrix, amino acid metabolism/transport, immunity, and/or steroidogenesis (see Table 2 for primers; Table 3 for accession numbers).

**TABLE 2:** Genes of Interest and their corresponding primers used in qPCR reaction. Annealing temperature for all primers was 60°C.

		
Gene ID	Primers	Amplicon length in bases
2OG-Fe(II)	F: GGA​GTT​TTC​AGG​TGG​TGA​GC	196
	R: ATT​GCG​GAC​TTT​TGA​TGA​CC	
17βHSD12	F: AGT​CCA​GAT​TTT​CTT​GCA​ACC​A	226
	R: TAG​ACT​TCA​GTG​GTG​GGC​AG	
17βHSD14	F: TGC​ACC​CTT​TGT​TGT​GAC​AT	209
	R: GAT​GGC​ATC​CTC​CAG​AAA​GA	
COL1A2	F: GATCCCAACAACGGCTGC	150
	R: TTC​ACT​AAA​CCA​TTC​TGC​TCC​G	
COL4A1	F: GCC​AGG​CAA​GTG​ACT​TCT​TA	199
	R: GTT​CCT​CCC​AAA​CAG​CAA​GT	
C3	F: TTA​TCA​TGG​TCC​TGG​GTG​CT	208
	R: GCG​TCA​AAC​TCG​AAC​GTT​TT	
CYB561	F: GGA​GAA​GCG​CAC​TGG​TCT​A	157
	R: GCC​TCC​ATT​CTG​CTG​TCA​TG	
FTH1	F: GAG​CTT​GAC​TCG​TCC​TCC​AC	197
	R: AGG​AAT​CCA​TCA​ACC​AGC​AG	
GLUL	F: TCG​TCT​ATC​TTC​AAG​GTA​GCC​C	157
	R: CCC​CAA​GCA​AGG​ACA​AGA​CT	
Heph	F: ACA​AGA​TGT​TTG​ACG​TGG​GC	232
	R: CGT​CTT​TGT​CAA​CTC​GTG​CA	
MCT2	F: TTG​GTT​ACG​GTG​TAG​GGG​AG	217
	R: GCA​AAG​ACT​TCT​AGC​GAG​CC	
MCT3	F: TGG​TTG​GTT​TGT​ATC​GCT​GC	167
	R: AGC​GGG​CCA​AAT​AGA​TAC​GA	
MCT10	F: ATC​TCA​TGC​CCG​TAT​GTA​GAC	151
	R: CCC​ATG​TCG​GCA​ACT​CTA​AT	
MYS	F: CGC​AAA​GAC​CTA​GAC​AAC​CG	209
	R: AGG​GAT​GTG​TGA​CTG​TGG​AG	
OAT	F: CCT​CGG​TGG​TAT​CTG​TGG​TT	120
	R: CCA​AGC​CGC​TAT​AAT​CAT​ATG​GG	
SLC31A2	F: TGA​AAC​AAA​GGC​TGA​GAG​AAG​A	121
	R: TAG​CCA​ACC​TCG​TGT​ATG​CA	
SLC39A14	F: GAT​CAA​TGT​CAA​ACA​CTG​TCA​TCA	150
	R: CTT​CAT​CAA​CAC​CTG​CTC​CA	
SLC8A3	F: CGG​CGT​GTT​TGT​TAT​TCT​GC	241
	R: GTA​CGT​GAC​CAC​ATT​CGA​GC	
TaDH	F: CAC​GAC​CCC​CAT​GAT​GTA​GT	178
	R: GGT​AGA​ACA​AAG​GCC​GTT​CA	
TF	F: TTT​TAC​AAT​GAA​AAC​GTC​TCA​AAC​TT	163
	R: GGC​AAG​TGC​TAT​CCC​AAG​TC	
TNR	F: CTACCGTCTTCAATGCATCCC	225
	R: AGAATGAGTGGGCAATGCATC	
TRPA1	F: TGA​AAC​CAT​GCA​TCG​CTC​AT	158
	R: TGA​AAG​ACA​TGC​AGA​CAC​AGC	

**TABLE 3:** Gene of interests (GOIs) and associated *Exaiptasia diaphana* accession numbers.

Putative gene	Gene abbreviation	Homolog accession #
2-oxoglutarate and iron-dependent oxygenase domain-containing protein 2	2OG-Fe (II)	XP_020905799
17-beta-hydroxysteroid dehydrogenase 12-like	17βHSD12	KXJ12187
17-beta-hydroxysteroid dehydrogenase 14-like	17βHSD14	KXJ20962
Collagen alpha-2 (I) chain	COL1A2	KXJ29536.1
Collagen alpha-1 (IV) chain	COL4A1	KXJ15297.1
Complement C3	C3	KXJ11955.1
Cytochrome b561	CYB561	KXJ17631.1
Glutamine synthetase	GLUL	AAR36878.1
Hephaestin-like	HEPH	XP_020906986.1
Integrin beta-PS	MYS	KXJ28340.1
low affinity copper uptake protein 2	SLC31A2, CTR2	XP_020892422.1
Monocarboxylate Transporter 2	SLC16A7, MCT2	XP_020903028
Monocarboxylate Transporter 3	SLC16A3, MCT3	XP_028513916
Monocarboxylate Transporter 10	SLC16A10, MCT10	KXJ27932.1
ornithine aminotransferase, mitochondrial-like	OAT	XP_020909112
Serotransferrin-like	TF	XP_020907010.1
Sodium/calcium exchanger 3	SLC8A3	XP_020912295
Soma ferritin-like	FTH1	XP_020908035.1
Tauropine dehydrogenase	TaDH	XP_020915529.1
Tenascin-R	TNR	KXJ05169.1
Transient receptor potential cation channel subfamily A member 1-like	TRPA1	KXJ26138.1
Zinc transporter ZIP14	SLC39A14, ZIP 14	KXJ16968.1

### Transcriptional responses of GOIs

**FIGURE 1:**
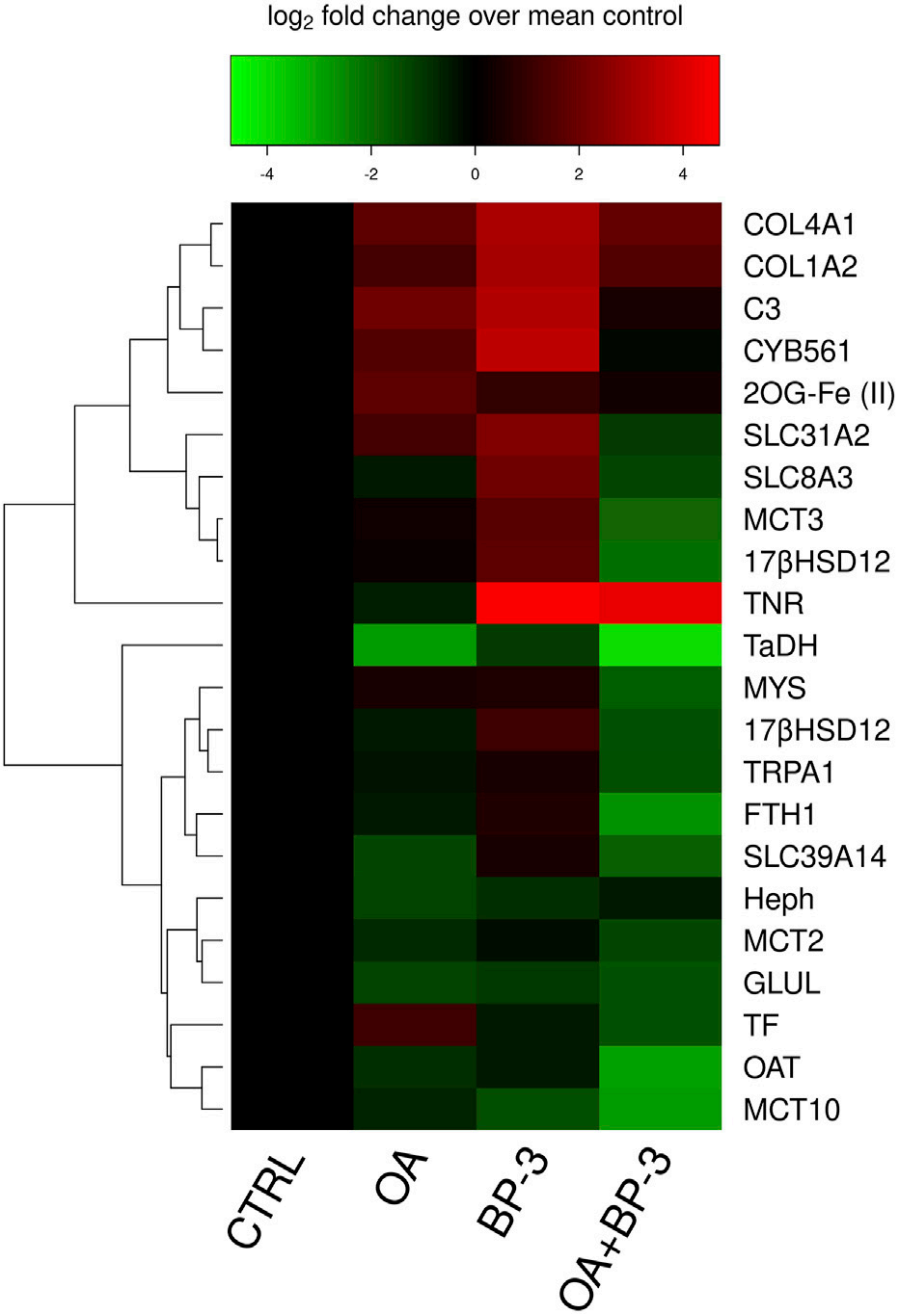
Heatmap of differential expression for all 22 genes of interest in each treatment.

All 22 GOIs exhibited significant changes in transcription to OA conditions, and/or BP-3 exposure, and/or the interaction of OA and BP-3 (Figure 1: heatmap Table 4: *p*-values of significant responses). Twenty GOIs had significant responses to OA and two GOIs (TF, TNR) were not significantly responsive to OA. Fourteen GOIs were responsive to BP-3 exposure while the remaining eight GOIs were not responsive to BP-3 (2OG Fe(II), SLC39A14, MCT2, MCT3, SLC8A3, 17βHSD14, TRPA1, SLC31A2). Five GOIs did not have significant interactions between OA conditions and BP-3 exposure (MCT2, MCT10, GLUL, TF, TNR).

**TABLE 4:** *p*-values for each GOI in the presence of OA, BP, or any significant interaction between OA and BP-3. Values in bold are significant.

Gene	OA	BP-3	Interaction
SLC39A14	**<0.001**	0.9341	**0.0213**
tadh	**<0.001**	**<0.001**	0.8246
TF	0.8556	**<0.001**	**<0.001**
FTH1	**<0.001**	**<0.001**	**<0.001**
2OG-Fe II	**0.0161**	0.3359	**<0.001**
TRPA1	**<0.001**	**0.0101**	**<0.001**
MYS	**<0.001**	**<0.001**	**<0.001**
TNR	0.3284	**<0.001**	0.9341
SLC8A3	**<0.001**	0.1243	**0.0037**
MCT10	**0.0043**	**<0.001**	0.1891
MCT3	**0.0021**	0.4997	**<0.001**
CYB561	**0.0093**	**0.0183**	**<0.001**
COL1A2-11	**0.0089**	**<0.001**	**<0.001**
COL4A1-11	**<0.001**	**<0.001**	**<0.001**
MCT2	**0.0227**	0.2464	0.6405
HSD14	**<0.001**	0.9436	**0.0046**
HSD12	**<0.001**	**0.024**	**<0.001**
C3	**0.0173**	**<0.001**	**<0.001**
GLUL	**<0.001**	**0.0057**	0.0843
OAT	**<0.001**	**<0.001**	**<0.001**
Heph	**0.0042**	**0.0042**	**<0.001**
SLC31A2-1	**<0.001**	0.9745	**<0.001**

### Docking simulations

**FIGURE 2:**
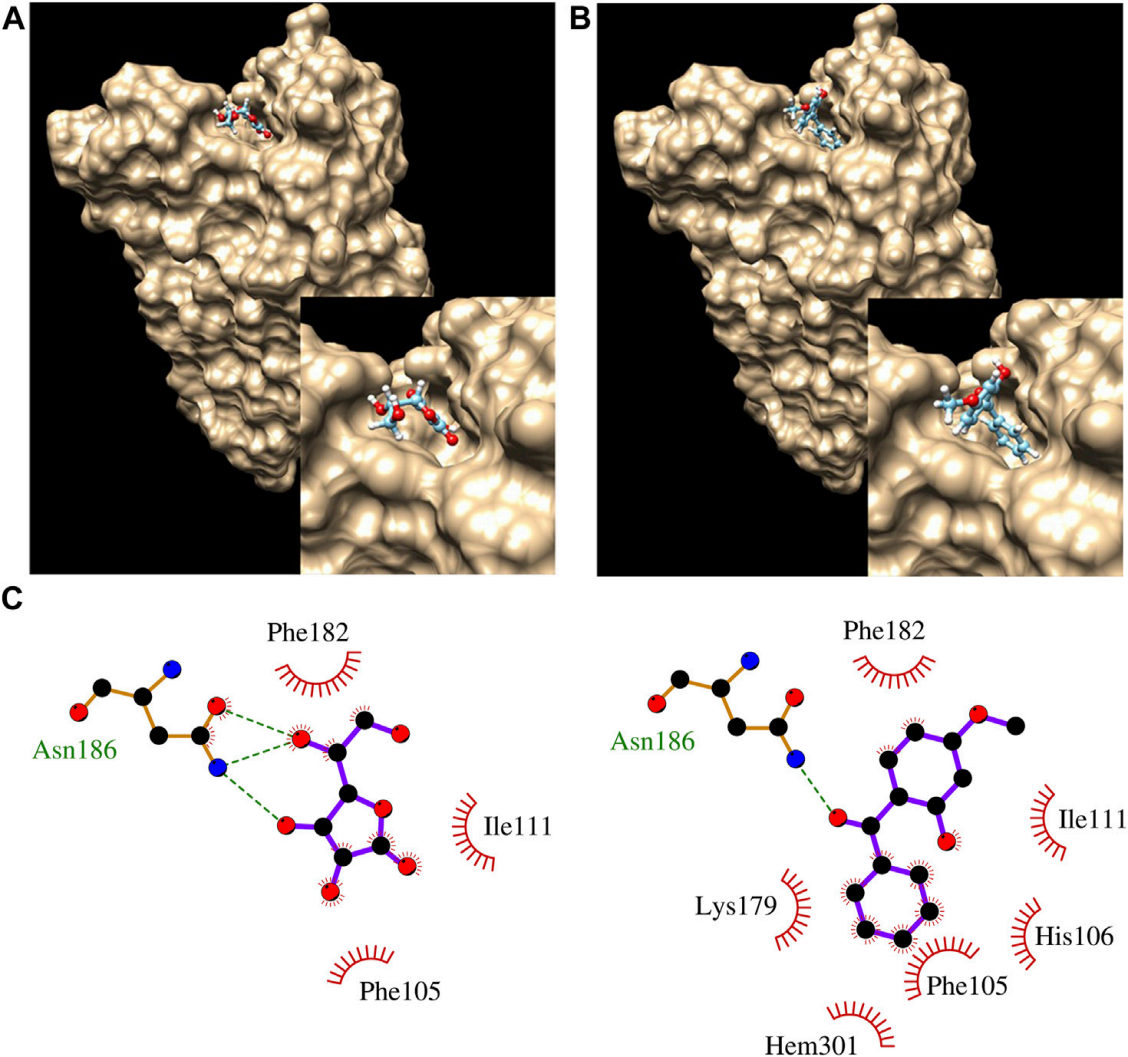
Docking results showing ascorbate **(A)** and BP3 **(B)** bound to CYB561. The inset pic at bottom right corner of A and B show closeup of ascorbate and BP3, respectively in binding pocket. **(C)** LigPlot analysis of ascorbate (left) and BP3 (right), showing interactions with nearby residues. Hem301 is a nearby heme group.

*In silico* modeling was performed on MCT2, MCT3, MCT10, and CYB561. MCT2 and MCT3 docking simulations included binding affinities for lactate which is the recognized molecule of transport for proton-dependent transporters. MCT1 was modeled as a comparison since it is the most-well characterized proton-dependent transporter with known inhibitors (BAY8002 and 7ACC2). Docking simulations with MCT10 included amino acids with aromatic side chains which are the recognized molecules of transport (Table 5). Docking simulations of CYB561 reveal there are seven amino acid side chains that interact with BP-3 (Figure 2). By comparison, CYB561 has 4 amino acids interacting with ascorbate (see Figure 2). Table 5 provides a comparison of the binding affinities for BP-3 and ascorbate.

**TABLE 5:** *In silico* binding affinities (kcal/mol). BY8002 and 7ACC2 are recognized inhibitors of MCT1. Phe, Tyr, and Trp are the aromatic amino acids normally transported by MCT10. *actual structure used instead of homology model. Transporter orientation towards the cytosolic side of the plasma membrane are labeled “in”. Transporter orientation towards the extracellular side of the plasma membrane are labeled “out”. Values in bold are significant.

Receptor	Ligand					Template
	BP3 (protonated)	BP3 (deprotonated)	L-lactate	BAY8002	7ACC2	
**MCT1-in**	−8.202	−8.362	−3.852	−9.472	−10.555	7cko*
**MCT1-out**	−7.343	−8.088	−4.027	−10.679	−8.247	7ckr*
**MCT2-in**	−8.534	−8.733	−3.784			7cko
**MCT2-out**	−7.311	−7.356	−3.89			7ckr
**MCT3-in**	−7.681	−7.667	−3.811			7cko
**MCT3-out**	−6.531	−6.854	−3.591			7ckr
			**Phe**	**Tyr**	**Trp**	
**MCT10-in**	−6.829	−6.931	−6.166	−6.173	−6.659	7cko
**MCT10-out**	−7.458	−7.551	−6.201	−6.264	−7.279	7ckr
			**ascorbate**			
**CYB561 (cytosolic)**	−4.69	−4.705	−4.79			4o79*
**CYB561 (noncytosolic)**	−6.743	−6.938	−4.518			4o7g*

### Categorizing synergistic, antagonistic, or additive effects

**FIGURE 3:**
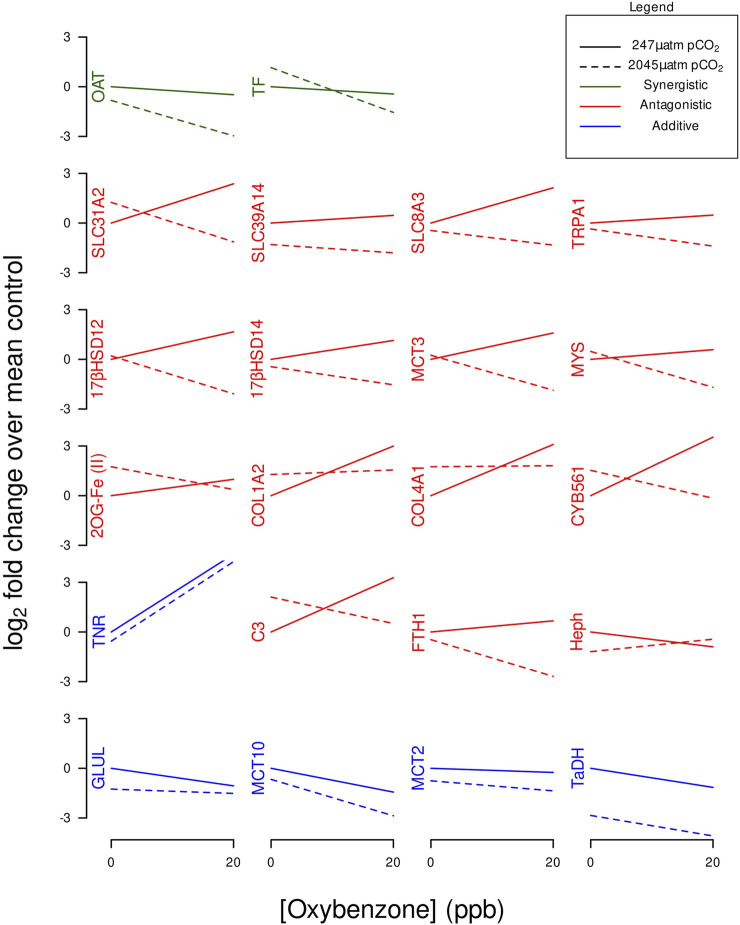
Interaction plots of GOI expression profiles categorized by synergistic, antagonistic, or additive effects.

Figure 3 identifies which GOIs had synergistic, antagonistic, or additive effects. Table 4 identifies which GOIs had significant interactions. Two GOIs (TF and OAT) had significant synergistic transcriptional interactions on their respective gene networks (see Table 4 and Figure 3). Fifteen GOIs had significant transcriptional interactions with antagonistic effects (see Table 4 and Figure 3). Five GOIs (GLUL, MCT10, MCT2, TaDH, TNR) exhibit additive responses when BP-3 is present in acidified seawater (see Figure 3).

## Discussion

### pH in experimental setup

At the beginning of 1 month of acclimation, the pH was not measured. After 1 month of acclimation, the pH of 8.25 was recorded at the beginning of the experiment. The pH of the local seawater is typically between 7.8–8.0 (Murray et al., 2015; Onthank et al., 2021). Differences between the normative pH range of local waters and the pH measured at the beginning of the treatments is likely due to 1) anemone’s algal symbionts favoring carbon fixation (removal of CO_2_) under the laboratory conditions during the 1 month of acclimation; 2) bubbling of air into the control tank helped drive off excess pCO_2_ from the local waters. Coral Reefs are known to experience fluctuations in pH of 0.5 units (Birkeland et al., 2008).

The pH range used in this investigation was important because it spanned the pKa of BP-3. Speciation of a compound is determined by pH and the compound’s pKa. If a compound’s pKa is the same as the pH, its ionized and unionized molecules are at equal concentrations (Xin et al., 2021). BP-3′s pKa is 7.6 (Fontanals et al., 2010). The laboratory-controlled acidic conditions of pH 7.5 favored a shift towards a protonated form of BP-3 but there was not a significant difference in the binding affinities of protonated *versus* deprotonated forms of BP-3 (see Table 5).

### Functional significance of expression profiles

#### Monocarboxylate transporters and links to intracellular pH

**FIGURE 4:**
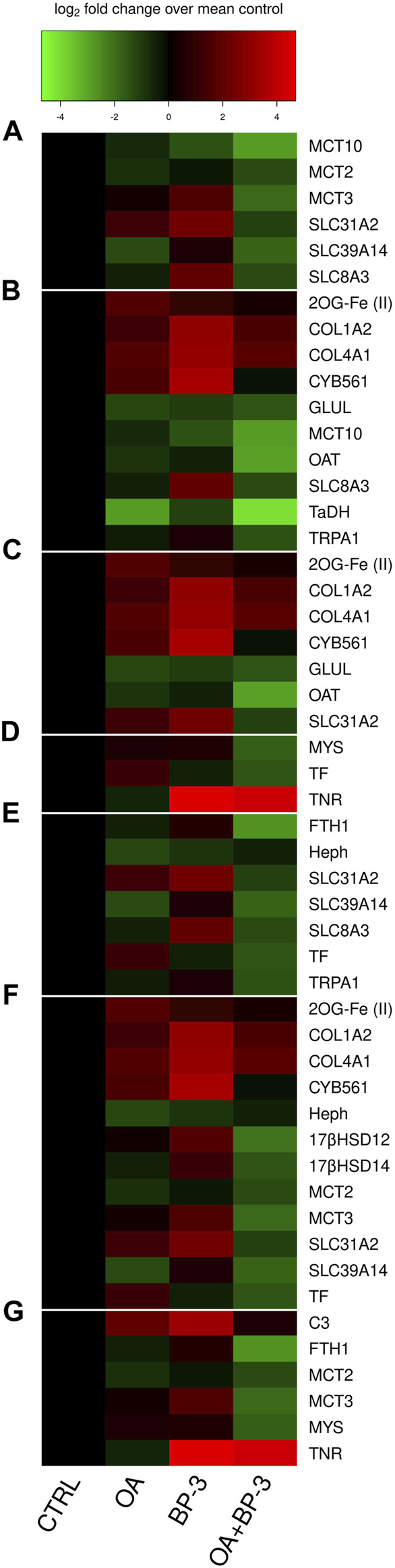
Categorized heatmaps of GOIs exhibiting significant differences in expression in response to OA and/or BP-3 exposure. Group **(A)** Soluble carrier proteins GOIs: SLC31A2, SLC39A14, SLC8A3, MCT3 (SLC16A3), MCT2 (SLC16A7), MCT10 (SLC16A10). Group **(B)** GOIs associated with amino acid synthesis, reservoirs, and metabolism. Group **(C)** GOIs associated with Collagen synthesis. Group **(D)** Extracellular matrix GOIs. Group **(E)** ion transport GOIs. Group **(F)** 12 GOIs with functional links to steroidal hormones. Group **(G)** Immune-related responses.

While intracellular pH was not directly measured in this investigation, some expression profiles suggest intracellular pH was lowered by exposure to OA. MCT expression is known to be influenced by intracellular pH due to extracellular pH (Gopal et al., 2004). MCTs play a role in the maintenance of intracellular pH which is critical for physiological function (Casey et al., 2010; Halestrap, 2013). Monocarboxylate transporters function as sensors and regulators of pH (Casey et al., 2010; McBrian et al., 2013). MCT 1,2,3, and 4 are symporters of monocarboxylates and protons (1:1 ratio) and the direction of transport across plasma membrane is determined by the relative intra- and extracellular pH (Iwanaga and Kishimoto, 2015). Proton-dependent MCTs are stimulated by decreasing the pH from 8 to 6 and with lowered pH, the Km value (Michaelis constant) for MCTs decreases thereby improving efficiency for binding and transport (Halestrap and Meredith, 2004; Halestrap, 2013). MCT 1&2 pump compounds and protons in one direction while MCT 3&4 pump the same compounds and protons in the opposite direction (Yoshida, 2021). The process of different MCTs coordinating transport of the same compounds in opposite directions is commonly described as metabolic symbiosis (Payen et al., 2020). The expression profiles of MCT2 (SLC16A7) and MCT3 (SLC16A3) in the OA treatment is consistent with the concept of metabolic symbiosis as each would be pumping in opposite direction (see Figure 4A). Data herein is consistent previously published studies demonstrating downregulated transcription of proton-dependent MCTs under acidic conditions (Casey et al., 2010) (see Figure 4A). The gastrovascular pH is known to be more acidic than surrounding seawater (Cai et al., 2016; Bove et al., 2020). Since whole animal homogenate was used in this investigation, future studies should investigate *in situ* hybridization of MCT2 vs. MCT3 to determine which tissues are most likely to utilize which MCT. Calcium ATPase have previously been the focus of identifying potential proton exchangers that help regulate pH in stony corals (Zoccola et al., 2004; Tambutté et al., 2011). The responsiveness of the proton-dependent MCTs presented herein offers another potential regulator of intracellular pH to consider in future studies. Coupled with the responsiveness of SLC8A3 (Na+/Ca^2+^ exchanger), these MCTs may provide an alternative mechanistic option for modulating Ca^2+^ and intracellular pH in stony corals.

#### Links to amino acid synthesis, reservoirs, and metabolism

The following amino acids are associated with expression profiles generated in the investigation: glutamine, glutamic acid, proline, hydroxyproline, amino acids with aromatic side chains, and the non-proteinogenic amino acids taurine and tauropine.

Glutamine synthetase (GLUL) is required to convert glutamic acid into glutamine. Glutamine synthetase regulation is pH-sensitive (Frieg et al., 2021). The downregulated transcription of glutamine synthetase (Figure 4B) suggests lower cellular demand for glutamine allowing glutamate to be utilized/shifted towards other metabolic demands. Under intracellular acidic conditions, four biochemical reactions occur: 1) Glutamine is deamidated by direct hydrolysis (Li et al., 2010), 2) the interconversion of glutamate and α-ketogluarate by glutamate dehydrogenase (GDH) is driven in the direction of glutamate synthesis. 3) the interconversion between glutamate and glutamate-γ-semialdehyde (GSA) is driven towards GSA synthesis which is an intermediate precursor of proline. 4) the interconversion of ornithine and GSA relies on ornithine-δ-aminotransferase (OAT) and the reaction is driven in the direction of GSA (a proline precursor). Under acidic conditions, these four biochemical reactions all move in the direction of proline synthesis. Glutamate metabolism and proline synthesis are functional links between the TCA and urea cycles. Proline plays an important role in regulation of synthesis of ornithine, arginine, polyamines, glutamate and collagen (Karna et al., 2020). In this investigation, there is downregulated transcription of OAT because the cellular demand for converting ornithine into GSA is lessened because glutamate abundance is already elevated in the acidic environment (see Figure 4B). When OAT demand is upregulated, there is increased proline catabolism (Albaugh et al., 2017).

The upregulated transcription profiles of collagen chains I and IV (see Figure 4B) are consistent with an increased metabolic demand for proline synthesis since proline is a major component of collagen. Newly synthesized proline normally accumulates in cytoplasm where it helps buffer cytosolic pH (Meena et al., 2019). If cells are experiencing an intracellular acidic pH, then upregulated synthesis of proline won’t offset intracellular acidic pH because newly synthesized proline is utilized in collagen synthesis. The upregulated transcription of collagen chains (COL1A2; COL4A1) (see Figure 4B) suggests increased cellular demand for synthesizing proline which must then be post-translationally modified to produce the hydroxyproline residues needed for collagen. Both proline and hydroxyproline have important metabolic and physiological roles (Li and Wu, 2018).

Tauropine dehydrogenase (TaDH) is responsible for the interconversion of two non-proteinogenic amino acids Tauropine and Taurine. The abundance of Tauropine typically correlates with the concentration of taurine (derivative of methionine or cysteine) (Linsmayer et al., 2020). The expression profile of TaDH (see Figure 4B) reveals Tauropine and Taurine are relevant in the context of OA where the interconversion reaction is driven towards Taurine accumulation. Tauropine is produced under hypoxic (fermentation) conditions. There is growing recognition of the importance of fermentative metabolism for cnidarian biology (Linsmayer et al., 2020). Fermentation will increase demand for proton-dependent MCTs to pump lactate out of cells. Taurine levels increase in pathological conditions where its antioxidant capabilities help to downregulate of inflammatory mediators (Voskanyan et al., 2014). Taurine’s buffering capacity reduces the negative impact of lactate by modulating intracellular free Ca^2+^ during fermentation conditions. Taurine lowers intracellular Ca^2+^ levels by inhibiting calcium influx from calcium channels including the reverse mode of Na+/Ca^2+^ exchanger (SLC8A3) (Li et al., 2005). Calcium influx can also occur through TRPA1 which is responsive to taurine (Redmond et al., 2014; Meents et al., 2019). Under acidic conditions, glutamate synthesis is elevated which elevates the intracellular Ca^2+^ concentration. Taurine reduces glutamate-induced elevated Ca^2+^ concentration (Ripps and Shen, 2012). Expression profiles for both SLC8A3 and TRPA1 are downregulated which reaffirms a reduced cellular demand for Ca^2+^ influx (see Figure 4B).

MCT10 transports amino acids with aromatic side chains (Imagawa et al., 2004; Yoshida, 2021). Cnidarians import essential amino acids (including phenylalanine and tyrosine) directly from symbiotic algae (Wang and Douglas, 1999) and tyrosine is known to be can be transferred to marine invertebrate larvae via the food chain (Heyland and Moroz, 2005). MCT10’s recognized function offers a potential mechanistic explanation for how cnidarians import the amino acids referenced in these previous studies. It has also recently been discovered that another monocarboxylate transporter MCT7 will transport taurine, a non-proteinogenic amino acid (Higuchi et al., 2022).

### Links to collagen synthesis and the extracellular matrix

The four major classes of proteins associated with the ECM are structural proteins, glycoproteins, glycosaminoglycans and proteoglycans, and matricellular proteins (Jones et al., 2015). Seven GOIs (COL1A2, COL4A1, CYB561, 2OG-Fe(II), GLUL, OAT, SLC31A2) are associated the collagen synthesis (Figure 4C). Collagen belongs to the structural class of the ECM proteins. The upregulated transcription of collagen chains I & 4 (COL1A2; COL4A1, respectively) suggests increased cellular demand for proline which must be post-translationally modified to produce hydroxyproline for collagen. Proline synthesis occurs through the glutamate cycle (requiring GOI: GLUL) and/or through modifications of ornithine (requiring GOI: OAT). Ascorbate is needed to hydroxylate proline residues in collagen processing which also requires 2-oxoglutarate oxygenase with Fe^2+^ as cofactor (GOI: 2OG-Fe(II)) and cytochrome b561 (GOI: CYB561) to bind ascorbate (Peterkofsky, 1991; Loenarz and Schofield, 2008; Berczi and Zimanyi, 2014). Cu^2+^ uptake is stimulated by ascorbate, which acts as a Cu^2+^ reductant (Schweigel-Röntgen, 2014). The upregulation of SLC31A2 (aka: CTR2) is consistent with expression profiles of genes associated the collagen synthesis and an increased metabolic demand for ascorbate (see Figure 4C).

Collagen is upregulated in wound healing (Albaugh et al., 2017). Under acidic conditions, collagen synthesis is upregulated and the proline incorporated into collagen removes it from the metabolic pool (Chalecka et al., 2021). Mechanical properties of collagen formed under alkaline conditions are better than collagen formed under acidic conditions (Zhang et al., 2022). Data herein cannot discern whether upregulated collagen synthesis is due to demand for additional collagen or replacement of existing collagen.

Three GOIs (Mys, TNR, TF) have functional associations with the extracellular matrix (ECM) (Figure 4D). Integrins are trans-membrane proteins that facilitate interactions between cells and ECM (Gotwals et al., 1994; White et al., 2004). Tenascin (TNR) (aka: ryncolin-4) has a FReD (fibrinogen domain), associated with wound healing, and known to be responsive to acidified seawater (Tran et al., 2004; Moya et al., 2012). TNR belongs to the matricellular class of ECM proteins. Serotransferrin (TF) is an iron-binding glycoprotein delivering iron to cells (Gkouvatsos et al., 2012). TF belongs to the glycoprotein class of ECM proteins. MCT 1-4 require and bind directly to basigin or embigin, single-pass transmembrane glycoproteins that are necessary for MCT activity (Halestrap, 2013; Muramatsu, 2016). Integrins strongly interact with glycoproteins (basigin or embigin) to facilitate integrin signaling inside of cell as well as the glycoprotein’s activity (Muramatsu, 2016). During an acute phase of wound healing, a drop in pH can cause hypoxia and increased production of lactic acid (Jones et al., 2015). Expression profiles of the GOIs associated with collagen synthesis and the other ECM transcripts are consistent with previous cnidarian research demonstrating CO_2_-driven acidification enhances synthesis of the extracellular matrix (Moya et al., 2012).

### Links to ion transport

Seven GOIs (FTH1, Heph, SLC31A2, SLC39A14, SLC8A3, TF, TRPA1,) function in cation transport (Figure 4E). Two GOIs (SLC8A3, TRPA1) transport Ca^2+^. The Na+/Ca^2+^ exchanger (SLC8A3) exchanges one Ca^2+^ for three Na+. The Na+/Ca^2+^ exchanger functions in both import and export of Ca^2+^ for maintenance of intracellular Ca^2+^ homeostasis, however the predominant mode is to export Ca^2+^ (Khananshvili, 2013; Barron et al., 2018). Transient receptor potential cation channel subfamily A member 1 (TRPA1) is a nonselective cation channel permeable to Ca^2+^, Na+, and K+. The TRPA1 channel is strongly regulated by both extracellular and intracellular calcium (Meents et al., 2019). While SLC8A2 and TRPA1 are linked to calcium transport, they are not exclusively linked to calcification since anemones are not calcifiers.

Three GOIs (Heph, FTH1, TF) function in iron binding/transport. Hephaestin, a transmembrane ceruloplasmin homolog involved with iron efflux and considered an important link between copper and iron metabolism (Vulpe et al., 1999; Lang et al., 2012). Serotransferrin (TF) is an iron-binding glycoprotein involved with iron influx to cells (Gkouvatsos et al., 2012). Ceruloplasmin and TF are known to interact as positive and negative controls in an acute phase response to perturbations of copper and iron metabolism (Golizeh et al., 2017). Hephaestin, a ceruloplasmin homolog has previously demonstrated acute responsiveness to exogenous copper (Morgan and Snell, 2006). Somaferratin (FTH1) functions in iron storage, detoxification, and innate immunity (Voolstra et al., 2009). Expression profiles for Heph, FTH1, and TF reveal the cellular demand for iron influx/eflux (TF expression) varied significantly with the presence/absence of BP-3 in OA conditions (see Figure 4E).

Intracellular concentrations of Cu^2+^ and Zn^2+^ are altered in response to OA. As a member of the SLC39 family, SLC39A14 is plasma membrane protein involved with Zn^2+^ influx and increasing the availability of cytosolic Zn^2+^ (Schweigel-Röntgen, 2014). Members of the SLC31 family act to regulate the intracellular Cu^2+^ concentration. SLC31A2 is mainly found in intracellular membranes of the late endosome and lysosome and is known to regulate intracellular Cu^2+^ concentration (Wee et al., 2013; Schweigel-Röntgen, 2014). Dysregulation of Zn^2+^ and Cu^2+^ are known to be associated with diseased conditions (Lynes et al., 2007; Schweigel-Röntgen, 2014).

#### Links to steroidal hormones

Twelve GOIs (2OG-Fe (II), 17βHSD14, 17βHSD12, COL1A2, COL4A1, CYB561, Heph, MCT2, MCT3, SLC31A2, SLC39A14, TF) have functional links to steroidal or tyrosine-based hormones (Figure 4F). BP-3 is recognized as an endocrine disruptor (Downs et al., 2016; LaPlante et al., 2018; Matouskova et al., 2020). *17*β*HSD14* and *17*β*HSD12* are associated with steroidogenesis. *17*β*HSD14* converts estradiol (E2) into estrone (E1); and testosterone (T) into androstenedione (A4), while *17*β*HSD12* performs the reverse reactions, E1 into E2, and A4 into T (Tarrant et al., 2003; Luu-The et al., 2006; Shikina and Chang, 2016; Salah et al., 2019). Proton-dependent MCTs are regulated by sex hormones (Felmlee et al., 2020). Solute carrier transporters SLC39A and SLC31A families are known to be regulated by estrogen (Schweigel-Röntgen, 2014). Estrogen can transcriptionally regulate synthesis of TF and ceruloplasmin (Golizeh et al., 2017), therefore it is highly probable Hephaestin (a homolog of ceruloplasmin) will respond in a similar manner to estrogen. Biosynthesis of collagen is subject to steroidal hormone regulation (Karna et al., 2020).

The variability in expression of these 12 GOIs reveals how BP-3 exposure influences their expression profiles in the presence/absence of OA conditions. Expression profiles of 17βHSD14 and 17βHSD12 in the presence of BP-3 are consistent with a previous investigation where there was upregulated transcription of both 17βHSD14 and 17βHSD12 in the presence of BP-3 (Morgan et al., 2022). However, upregulated expression of 17βHSD14 in response to BP-3 in this investigation was not statistically significant (see Table 4) suggesting the cellular demand for converting E2 into E3 was not significantly different from the control condition. Future studies investigating the reproductive status of anemones at the time of BP-3 exposure can be important to ascertaining the sensitivity of the anemone’s response to BP-3 alone *versus* in an environment of acidified seawater and BP-3. This will be particularly important in the abilities of these GOIs to detect endocrine disruption in a multi-stressor environment.

#### Links to immune responses

Six GOIs (FTH1, C3, MCT2, MCT3, MYS, TNR) have functional links to immune responses (Figure 4G). Cnidarian integrins are involved in multiple immunological cellular processes such as cell migration, differentiation, signal transduction, and wound repair (Miller et al., 2007; Knack et al., 2008; Shearer et al., 2012). Ferritins such as somaferritin (FTH1) are associated with innate immunity (Voolstra et al., 2009). C3 compliment is a recognized component of cnidarian innate immune system (Miller et al., 2007; Schwarz et al., 2008; Kvennefors et al., 2010; Palmer and Traylor-Knowles, 2012; Ocampo et al., 2015). Tenascin-R (TNR) is also known as ryncolin-4 is considered part of the innate immune system, involved with activating the Compliment pathway, and is upregulated in acidic conditions (OmPraba et al., 2010; Zúñiga-Soto et al., 2023). To be activated, proton-dependent MCTs need to bind immuno-globulin transmembrane glycoproteins (Muramatsu, 2016).

### Functional links to *in silico* modeling

*In silico* modeling demonstrates BP-3 favorably binds to MCT2, MCT3 with affinities much closer to recognized inhibitors (see Table 5). MCT10′s binding affinity to BP-3 is most similar to tryptophan (see Table 5) which suggests a plausible mechanism for importing BP-3 into a cell. BP-3 binding into the pocket of CYB561 facing the extracellular side of the plasma membrane suggests exogenous BP-3 may act as competitive inhibitor of ascorbate (see Table 5 and Figure 2). This conclusion is supported by the upregulated transcription of CYB561 (see Figures 1, 3). Structurally, Asn 186 functions by hydrogen bonding to the ligand; there are three putative hydrogen bonds to ascorbate and one hydrogen bond to BP-3. However, BP-3 has a greater surface area and is more hydrophobic compared to ascorbate, and the increase in van der Waals interactions between CYB561 and BP-3 explains the stronger binding affinity predicted by docking experiments. His 106, Lys 179, and the nearby heme group (Hem 301) are all involved in BP-3 binding, while these interactions are absent when ascorbate is bound. His 106 is highly conserved and known to participate in binding the two heme-b centers. In a previous study, a laboratory-induced F105W/H106E double mutation completely abolished the ascorbate-dependent redox activity of CYB561, and thus demonstrated His 106 is the single most critical residue for binding to the non-cytosolic side of the protein (Berczi and Zimanyi, 2014).

## Final conclusion

### Functional significance of synergistic, antagonistic, or additive interactions

Results herein reveal how synergistic, antagonistic, and/or additive responses potentially work in concert to produce cellular responses that might be described as homeostatic or adaptive.

While Serotransferrin (TF) had a significant synergistic interaction and Heph had a significant antagonistic interaction, both GOIs contribute to modulating the intracellular transcriptional demands for iron. TF is an iron-binding glycoprotein involved with iron influx (Castellano et al., 1994; Gkouvatsos et al., 2012). Hephaestin (Heph) is a transmembrane-bound homolog of ceruloplasmin involved with iron efflux and considered an important link between copper and iron metabolism (Vulpe et al., 1999; Lang et al., 2012). Ceruloplasmin and TF are known to interact as positive and negative controls in an acute phase response to perturbations of iron metabolism (Golizeh et al., 2017). These GOIs (TF and Heph) with significant but opposite interactions (see Figure 3) appear to represent the molecular underpinnings of iron homeostasis.

Under acidic conditions, the synergistic transcriptional response of OAT (reduce the biosynthesis of ornithine) coupled with the additive transcriptional response of GLUL (reduce the synthesis of glutamine) drive both pathways in the direction of proline synthesis. The biosynthesis of glutamate and proline can be linked to ornithine modifications (Majumdar et al., 2016).

Proline is a fundamental structural component of collagen. COL1A2, COL4A1, CYB561, and 2OG-Fe(II) are all involved with collagen synthesis and exhibit similar expression profiles under OA or BP-3 exposure (see Figures 1, 3). Multiple GOIs with similar significant interactions (i.e., antagonistic effects) involving the same process (i.e., collagen synthesis) appear to be in synchrony. Transcription of multiple GOIs needed for collagen synthesis represent the molecular underpinnings for the physiological response of adaptation to an acidic environment.

Antagonistic effects are representative of gene networks where the impacts of OA and BP-3 are working in opposition. Fifteen GOIs exhibit antagonistic effects when exposed to OA and BP-3 (see Figure 3). Twelve of these GOIs were downregulated when exposed to BP-3 in presence of acidified seawater reveals, whereas 14 GOIs were upregulated in the presence of BP-3 alone (see Figure 3). Under normal conditions, 17βHSD14 and 17βHSD12 perform functions that are opposite of each other. The expression profiles of these GOIs are consistent with a previous investigation where these GOIs exhibited transcriptional responses to an endocrine disruptive 20 ppb BP-3 exposure (Morgan et al., 2022). Future studies investigating longer exposures to BP-3 in an environment of OA will generate more comprehensive dose-response curves that will contribute to better characterization of the responsiveness of these GOIs and the other antagonistic responses.

### GOIs as drug targets

Influx and efflux transporters can act with intracellular metabolizing enzymes to regulate drug effects (Giacomini and Sugiyama, 2006; Goldstone, 2008). There is growing evidence that MCTs and solute carrier transporters (SLC8A3, SLC31A2) are responsive to various drugs (Halestrap and Wilson, 2012; Njiaju et al., 2012; Halestrap, 2013; Khananshvili, 2013; Iwanaga and Kishimoto, 2015). MCT 1&2 are drug targets for compounds acting as selective inhibitors (Payen et al., 2020; Wang et al., 2021). Anthropogenic chemicals can upregulate expression MCTs (Genter et al., 2002). In human health, MCTs are promising pharmacological targets and in cnidarian physiology, MCTs interact with xenobiotic chemical pollutants (Goldstone, 2008). Structures that are similar to ascorbate can inhibit enzymes usually working on ascorbate (Majamaa et al., 1986). TRPA1 is a promiscuous chemical sensor capable of responding to an unusually wide variety of chemically diverse compounds including many natural products and irritants, environmental chemicals, and common pharmaceuticals (Bautista et al., 2006; Jagadeesan, 2016; Meents et al., 2019). Tauropine dehydrogenase (TaDH) belongs to the ornithine cyclodeaminase (OCD)/mu-crystallin family. Substrate utilization of opine dehydrogenases is notoriously promiscuous (Linsmayer et al., 2020). Understanding the underlying molecular mechanisms of “drug-like” compounds such as BP-3 may lead to a more comprehensive interpretation of the expression profiles herein.

### Potential biomarkers of exposure to OA and BP-3

Developing transcriptional biomarkers in the laboratory will help to establish links between laboratory-based testing and adverse effects *in situ* (Depledge and Fossi, 1994). Development of biomarkers for cnidarian health are far behind biomarkers of human health. Using a reductionist approach, candidate biomarkers identified in this investigation can be valuable preliminary tools to future, more complex studies involving *in situ* monitoring. A first step biomarker development is to identify GOIs responsive to laboratory-controlled conditions on a single stressor (i.e., exposure to OA or BP-3). Eight GOIs representing collagen synthesis, zinc transport, transient receptor cation channel, and proteins containing a fibrinogen domain have previously demonstrated cnidarian responsiveness to OA (Moya et al., 2012; Wu et al., 2022). Three GOIs (C3, 17βHSD14, 17βHSD12) previously demonstrated responsiveness to BP-3 (Morgan et al., 2022). The only GOIs with known responsiveness to other anthropogenic chemicals include TNR, Heph, and select members of the solute carrier protein family (Morgan and Snell, 2006; Goldstone, 2008; Morgan et al., 2012).

A second step in biomarker development is to use the same GOIs to investigate responses under conditions of two stressors such as OA and BP-3 exposure. MCT 1-4 are recognized as potential biomarkers of human diseases (Felmlee et al., 2020). This investigation offers evidence (expression of proton-dependent MCTs in an acidic environment and *in silico* modeling of BP-3 binding to proton-dependent MCTs) which may represent a mechanism not previously recognized for modulating changes in intracellular pH in cnidarians. Both MCT2 and MCT3 discern differences in OA vs. normal pCO_2_ conditions which is consistent with MCT 2 and MCT 3 as pH regulators, but these MCTs cannot discern when BP-3 is present (see Table 4). MCT3 has a significant interaction between OA and BP-3 while MCT2 and MCT10 show no significant interactions between BP-3 and OA (see Table 4). MCT10 is downregulated in the presence of BP-3 (see Table 4) which could be environmentally relevant since increasing concentrations of free amino acids (including those with aromatic side chains) are known to occur in locations closer to anthropogenic sources (Yamashita and Tanoue, 2003). The value of CYB561 as a biomarker can be established by the OA/BP-3 and BP-3 expression profiles and confirmed by *in silico* docking of BP-3 which binds directly in the ascorbate binding pocket of CYB561 (see Figure 2).

A third step in the development of GOIs as biomarkers would be to investigate how they respond to multiple stressors such as OA, BP-3 exposure, and another stressor of global importance, thermal stress. GOIs associated with collagen synthesis and iron transport have previously demonstrated cnidarian responsiveness to elevated temperatures (Voolstra et al., 2009; DeSalvo et al., 2010; Kenkel et al., 2011). Critics cite multiple stressors causing mixed effects as being a vulnerable weakness for interpreting complex real-world conditions (Deidda et al., 2021). Anthropogenic activities represent complex mixtures of multiple stressors (Jones, 2010; Wear and Thurber, 2015; Mitchelmore et al., 2019; Mitchelmore et al., 2021). Cnidarian biomarkers for sensing changes in intracellular pH or pesticide exposure are emerging (Morgan and Snell, 2002; Barott et al., 2017). A biomarker developed in the lab subsequently demonstrated detectable responses in field sampling (Morgan and Snell, 2002). Characterizing the GOIs as synergistic, antagonistic, and additive responses provides a systematic process for interpreting the effects of multiple stressors. GOIs with synergistic and additive responses represent candidate biomarkers of exposure. The collective antagonistic responses of COL4A1, COL1A2, CYB561, and 2OG-Fe (II) strongly suggests these GOIs should be considered candidate biomarkers of effect. The observed changes in expression profiles of GOIs associated with collagen synthesis coupled with *in silico* binding of BP-3 to CYB561 offers a mechanistic explanation that underpins higher order physiological responses of organisms restructuring their extracellular matrix in response to an acidic environment.

Candidate biomarkers responsive to different stressed conditions can also be sensitive to different timeframes of stressor exposure. Results herein correlate well with previous transcriptional studies of cnidarian responses to short-term acidified seawater exposures or an acute exposure to 20ppb BP-3 (Moya et al., 2012; Morgan et al., 2022; Woo and Yum, 2022). The GOIs in this investigation represent responses not detected in study of >8000 genes in adult *A. millepora* after a 28-day exposure to acidic seawater (similar to pH in this investigation) that stimulated >600 differentially expressed transcripts (Kaniewska et al., 2012). Future studies establishing dose-response curves for these GOIs should be productive in elucidating differences in short-term vs. long-term responses to OA, exposure to BP-3, and/or the interaction between OA conditions and exposure to BP-3.

Transcriptional responses are the foundational framework for identifying candidate biomarkers. Gene expression profiling can be used to detect sub-lethal stress responses, differentiate the contribution of different anthropogenic stressors, and access an organism’s condition in natural environments (Snell et al., 2003; Evans and Hofmann, 2012). Gene expression profiling provides a portal into responsive cellular processes that converge and culminate into altered physiology. Gene expression profiles reveal responses that precede translational activity. *In silico* modeling provides molecular details about the precise location of impact an exogenous chemical can have on a specific protein. Transcriptional responses coupled with *in silico* modeling offer new insight into how environmentally relevant exogenous anthropogenic chemicals impact specific proteins. Results herein are consistent with responses of SLCs known to be associated with transport of H+, Na+, Ca^2+^, small chain aliphatic hydrocarbons, and amino acids (both proteinogenic and nonproteinogenic). The solute carrier family of proteins deserves further exploration in the context of xenobiotic chemical pollution in an environment of OA. Venn et al. (2013) stated “It is only with a better mechanistic perspective of the response of coral physiology to environmental change that forecasts of the future of coral reefs under ocean acidification and climate change can be improved”. Results from this investigation offer seven distinctive elements: 1) The expression profiles of these 22 GOIs are representative of important cellular processes impacting non-calcifying cnidarians and 17 of the 22 GOIs have significant interactions between OA and BP-3. 2) Fifteen GOIs exhibit antagonistic interactions to the presence of BP-3 in an environment of acidified seawater. The compensatory actions to one stressor *versus* both stressors depend on the cellular process impacted. 3) Data herein provides a plausible mechanistic explanation for how BP-3 binds to CYB561 and stimulates upregulation of genes associated with collagen synthesis in an environment of OA; 4) Data herein offers a plausible mechanism for how cnidarians in an environment of OA can modulate intracellular pH through proton-dependent monocarboxylate transporters; 5) Identification of 5 GOIs that should be considered “drug targets” for binding exogenous BP-3; 6) Identifying 12 GOIs with recognized functional links to hormones that are also responding to BP-3 and OA represent new avenues to pursue in the context of endocrine disruption; 7) By characterizing the expression profiles of 22 GOIs in a laboratory-controlled multiple stressor environment, these GOIs are one step closer to being candidate biomarkers for *in situ* field testing. Servetto et al. (2021) succinctly articulated how an understanding the molecular mechanisms underpinning the physiological processes is critical to properly interpreting cnidarian responses to acidic pH and their ability cope in the Anthropocene. Interactive effects of global stressors (e.g., ocean warming and/or acidification) and local stressors (e.g., pollution) can be advanced with a better understanding of the transcriptional responsiveness of these GOIs in the presence of multiple stressors. These 22 GOIs offer new directions for future studies on the interactions of ocean acidification and xenobiotic chemicals in cnidarians as well as other phyla containing calcifying and non-calcifying taxa.

## Data Availability

The datasets presented in this study can be found in online repositories. The names of the repository/repositories and accession number(s) can be found in the article/Supplementary material.
